# Long-term results after transoral outlet reduction (TORe) of the gastrojejunal anastomosis for secondary weight regain and dumping syndrome after Roux-en-Y gastric bypass

**DOI:** 10.1007/s00464-024-10989-3

**Published:** 2024-06-24

**Authors:** Jonathan Lovis, Stefan Fischli, Francesco Mongelli, Julia Mühlhäusser, Patrick Aepli, Martin Sykora, Andreas Scheiwiller, Jürg Metzger, Jörn-Markus Gass

**Affiliations:** 1 Department of General Surgery, Cantonal Hospital of Lucerne, Spitalstrasse , 6000 Lucerne 16, Switzerland; 2 Department of Endocrinology, Cantonal Hospital of Lucerne, Spitalstrasse, 6000 Lucerne 16, Switzerland; 3 Department of Surgery, Regional Hospital of Lugano, Via Tesserete 46, 6900 Lugano, Switzerland; 4 Department of Gastroenterology, Cantonal Hospital of Lucerne, Spitalstrasse , 6000 Lucerne 16, Switzerland; 5 Department of General Surgery, Cantonal Hospital of Nidwalden, Ennetmooserstrasse 19, 6370 Stans, Switzerland; 6 Department of Health Sciences and Medicine, University of Lucerne, 6002 Lucerne, Switzerland

**Keywords:** Bariatric surgery, Roux-en-Y gastric bypass, Endoscopy, Secondary weight regain, Dumping syndrome, Transoral outlet reduction

## Abstract

**Background:**

Bariatric surgery has been proven to be the most effective therapy for obesity and Roux-en-Y gastric bypass (RYGB) is one of the most commonly performed procedure. However, weight regain and dumping syndrome occur over time. The transoral outlet reduction (TORe) procedure using an endoscopic suturing device may be an option to treat these conditions. We aimed to analyze outcome parameters and long-term results for this endoscopic technique.

**Methods:**

A retrospective data analysis of patients who underwent TORe using an endoscopic suturing system at our institution from January 2015 to December 2020 was performed. A total of 71 subjects were included. Forty-five patients received the intervention for weight regain, 9 for dumping syndrome and 17 for both. The primary endpoint was weight stabilization or weight loss for subjects with weight regain, and resolution of symptoms for those with dumping syndrome. Secondary endpoints were intraoperative complications, procedure time, length of hospital stay and diameter of gastrojejunal anastomosis 1 year post-intervention.

**Results:**

The median size of the gastrojejunal anastomosis was estimated at 30 mm before intervention, and after performing a median of 3 endoscopic sutures, the median estimated gastrojejunal anastomosis width was reduced to 9.5 mm. Eight perioperative complications occurred. Overall mean follow-up was 26.5 months. All interventions achieved weight stabilization or weight loss or resolution of dumping symptoms within the first 3 months, 98.2% at 12 months, 91.4% at 24 months and 75.0% at 48 months. In 22/26 subjects a persisting improvement of dumping syndrome was achieved.

**Conclusions:**

TORe is a safe and effective procedure in the treatment of patients with dumping syndrome after laparoscopic RYGB, the effect on weight stabilization is less significant. A prospective randomized trial should be conducted to compare the effects of TORe with other surgical methods like banding the gastrojejunal anastomosis.

Bariatric surgery has been proven to be the most effective therapy with excellent long-term results compared to best medical care [1]. Roux-en-Y gastric bypass (RYGB) is one of the most commonly performed procedures. With the increasing number of patients undergoing bariatric procedures the number of revisional surgeries is rising as well [2]. Although RYGB is a very effective procedure, weight regain occurs in a relevant number of patients, but the frequency and degree vary widely according to different studies and length of follow-up [3–6]. Reasons for weight regain are multifactorial and therefore a multidisciplinary approach is necessary. Malcompliance with dietary recommendations and a lack of exercise years after the initial procedure contribute to failure of weight loss maintenance [7]. Additionally, anatomical factors such as dilation of the gastrojejunal anastomosis or the gastric pouch and gastro-gastric fistula are well known to be risk factors not only for weight regain but also for dumping syndrome [8, 9].

Recent data shows that the diameter of the gastrojejunal anastomosis might contribute to decreased satiety followed by an increased caloric intake and an accelerated transport to the small bowel with subsequent dumping syndrome [10]. Except for lifestyle and dietary changes, increasing physical activity and using GLP-1 agonists, the only solution until a few years ago was surgery. However, revisional procedures are associated with a higher morbidity and mortality and bare limited efficacy compared to primary bariatric procedures [11–13].

The introduction and rising popularity of novel interventional endoscopic techniques like transoral outlet reduction (TORe) offers new possibilities for the treatment of weight regain and dumping syndrome. Such techniques have the benefit of reduced peri-procedural risks and lesser operative trauma, with a shorter recovery and quicker return to work and daily activities than traditional surgery [14–17]. Although interventional endoscopic suture systems are nowadays widely used and accepted, some questions remain unanswered concerning technical details like interrupted vs. non-interrupted sutures vs. purse-string suture or the optimal diameter of the gastrojejunal anastomosis.

The aim of the present study was to assess long-term outcomes of TORe in patients presenting with weight regain or insufficient weight loss and dumping syndrome after RYGB surgery.

## Materials and methods

### Data collection

A retrospective query from medical records of patients who underwent TORe using an endoscopic suturing system at our institution from January 2015 to December 2020 was performed. We included subjects with secondary weight regain, dumping syndrome or both after primary laparoscopic RYGB or conversions from sleeve gastrectomy to RYGB. In all procedures a linear stapler anastomosis according to the Lönroth technique with insertion of 35 mm of the stapling device was performed. Nearly all patients (*n* = 66) received a gastroscopy or a gastrografin swallow test to assess the anatomical situation concerning gastric pouch size and diameter of the gastrojejunal anastomosis.

We reviewed electronic medical records for demographic data (age, gender, height, weight, body mass index), comorbidities (diabetes mellitus, arterial hypertension, obstructive sleep apnea syndrome, hyperlipidemia, gastroesophageal reflux disease, arthralgia, depressive syndrome), operative data (gastrojejunal anastomosis size pre- and post-intervention and at one month postoperative, gastric pouch size, number of endoscopic sutures, procedure time, intraoperative complications), length of hospital stay, improvement of dumping syndrome, weight and BMI changes during follow-up, and if necessary re-intervention (endoscopic or surgical).

We defined weight regain as an increase > 10 kg from nadir, a BMI > 35 kg/m^2^ after weight loss, an increase > 25% EWL from nadir or a BMI increase > 5 kg/m^2^ from nadir after bariatric surgery [18]. Dumping syndrome included early and late dumping syndrome and was characterized by clinical symptoms such as sweating, tachycardia, vertigo, nausea, vomiting, abdominal pain and cramps that did not improve despite medical treatment or dietary intervention.

### Endoscopic procedure

All TORe procedures were performed by an experienced interventional endoscopist using the OverStitch™ suturing device (Apollo Endosurgery, Austin, TX, USA). Patients` written informed consent was obtained. A gastroscopy was performed under general anesthesia to visualize the esophagus and assess sizes of the gastric pouch and the gastrojejunal anastomosis. An overtube protection was used and the gastrojejunal anastomosis was prepared for suturing by coagulation with an argon beamer. Then the suturing device was introduced and 1–3 sutures were placed. We solely performed continuous sutures instead of purse-string suture. We aimed at an anastomosis diameter of 10 mm. After the suturing procedure, the patency of the anastomosis was controlled. Patients were routinely discharged at the same day of the intervention. All subjects were followed by experienced dietitians pre- and postoperatively, and underwent a routine gastroscopy with assessment of the anastomosis’ diameter one month post-intervention. Nowadays a control endoscopy is performed 6 months post-intervention. Patients were instructed to follow a strict nutritional regimen (ca. 1200 kcal progression diet), beginning with clear fluids (water, tea) for the first 24 h after the procedure, followed by a liquid diet (soup, yogurt) for 2 weeks and a pureed diet for another 4 weeks. Standard follow-up visits were planned at 4 weeks, 3 months, 6 months and 1 year after the procedure.

### Outcome parameters

The primary endpoint was weight stabilization or weight loss (for subjects with weight regain) and symptoms resolution for subjects with dumping syndrome.

Secondary endpoints were intraoperative complications classified according to Clavien-Dindo, procedure time, length of hospital stay, duration of follow-up and diameter of gastrojejunal anastomosis 1 year after intervention [19].

### Statistical analysis

Due to group heterogeneity concerning indication for endoscopic treatments in all subjects, descriptive statistics were presented as absolute number and percentage for categorical variables, while continuous variables were presented as mean and standard deviation (SD). A Kaplan–Meier curve analysis was performed to assess patients who achieved weight loss or maintained initial weight over the time. Statistical analysis were performed on MedCalc® Statistical Software version 20.008 (MedCalc Software Ltd, Ostend, Belgium; https://www.medcalc.org; 2021).

## Results

### Patient characteristics

Over the study period, a total of 71 subjects were included in the study. The indication for TORe was weight regain in 45 (63.4%) subjects, dumping syndrome in 9 subjects (12.7%) and both weight regain and dumping in 17 (23.9%) subjects. Participants were divided into three groups according to the above-mentioned indication for the endoscopic procedure. Mean age was 39.8 ± 10.9 years, 61 (85.9%) patients were female and overall mean BMI was 33.7 ± 6.6 kg/m^2^ at baseline. Diabetes mellitus was present in 10 (14.1%) subjects, hypertension in 24 (33.8%), OSAS in 17 (23.9%), hyperlipidemia in 6 (8.4%), GERD in 13 (18.3%), arthralgia in 26 (36.6%) and depressive syndrome in 5 of them (7.0%). RYGB was performed as a primary procedure in 55 (77.5%) subjects. Eight patients underwent a revisional surgery after RYGB. One patient underwent revisional surgery because of anastomotic leakage. Another patient underwent a conversion into a bilio-pancreatic diversion due to secondary weight regain and persistence of dumping symptoms. Two patients underwent a revision of the gastrojejunal anastomosis due to secondary weight regain. Finally, four patients underwent a distalization of the jejunojejunal anastomosis due to secondary weight regain. Five patients underwent a conversion procedure from gastric banding to RYGB. Indication for one patient was slipping of the gastric banding and subsequent obstruction. Another patient suffered from reflux symptomatic as well as secondary weight regain. The reason for revisional surgery for three other patients was secondary weight regain. Three further subjects underwent a conversion procedure from sleeve gastrectomy to RYGB for secondary weight regain. Details about subjects` categories are reported in Table 1.

**Table 1:** Patient demographics and clinical characteristics

Indication to EP Characteristics	Metabolic *n* = 45	Dumping *n* = 9	Metabolic and dumping *n* = 17
Median age, years (IQR)	39.0 (29.7–48.0)	46.0 (39.5)	38.0 (28.7–46.2)
Female gender, *n* (%)	38 (84.4)	8 (88.9)	15 (88.2)
Baseline BMI, kg/m^2^ (IQR)	34.5 (30.6–38.2)	24.8 (22.9–27.8)	32.8 (30.0–35.7)
Diabetes, *n* (%)	8 (17.8)	0	2 (11.8)
Hypertension, *n* (%)	15 (33.3)	3 (33.3)	6 (35.3)
OSAS, *n* (%)	12 (26.7)	2 (22.2)	3 (17.6)
Hyperlipidemia, *n* (%)	3 (6.7)	2 (22.2)	1 (5.9)
GERD, *n* (%)	7 (15.6)	3 (33.3)	3 (17.6)
Recurrent arthralgia, *n* (%)	19 (42.2)	2 (22.2)	5 (29.4)
Depressive syndrome, *n* (%)	2 (4.4)	2 (22.2)	1 (5.9)

Values are presented as median with interquartile range (IQR) or absolute number with percentage in parentheses

*EP* endoscopic plication, *BMI* body mass index, *GERD* gastroesophageal reflux disease, *OSAS* obstructive sleeping apnea syndrome

### Outcome of endoscopic procedure

Mean time between bariatric surgery and TORe was 80.0 ± 45.6 months: 77.7 ± 45.0 months in the weight regain group, 65.9 ± 36.6 months in the dumping group and 93.5 ± 50.7 months in the weight regain and dumping group. All interventions (100% or 71/71 subjects) achieved weight stabilization or weight loss or resolution of dumping symptoms within the first 3 months, 100% (65/65 subjects) at 6 months, 98.2% (56/57 subjects) at 12 months, 95.3% (41/43 subjects) at 18 months, 91.4% (32/35 subjects) at 24 months, 80.8% (21/26 subjects) at 36 months and 75.0% (9/12 subjects) at 48 months.

The mean size of the gastrojejunal anastomosis was estimated at 27.9 ± 4.7 mm before intervention and, after performing a mean of 2.9 ± 0.8 endoscopic sutures, the mean estimated gastrojejunal anastomosis width was reduced to 9.5 ± 1.7 mm. Overall procedure time was 39.9 ± 10.8 min. and 8 (11.3%) perioperative complications occurred. (Table 2) Three subjects with post-interventional dysphagia due to peri-anastomotic edema required antiemetics and intravenous fluids (Clavien-Dindo Grade I), two cases of hematemesis were managed conservatively (Clavien-Dindo Grade I), two intraoperative lesions were managed endoscopically with application of clips (one lesion of the esophagus and one perforation at the blind end of the gastrojejunal anastomosis). One perforation which occurred directly at the anastomosis and became symptomatic 3 days post-operative, was closed with a laparoscopic suture. All complications occurred at the very beginning of the study period when the implementation of this technique was still new.

**Table 2:** Postoperative and follow-up results

Indication to EP Characteristics	Metabolic *n* = 45	Dumping *n* = 9	Metabolic and dumping *n* = 17	*p*
Outpatients, *n* (%)	42 (93.3)	8 (88.9)	14 (82.4)	0.432
Length of stay for inpatients, days (IQR)	1 (1–1)	1 (1–1.5)	1 (1–1)	0.609
Improvement of dumping syndrome, *n* (%)	–	9 (100)	13 (76.5)	0.121
Surgical revisions, *n* (%)	12 (26.7)	2 (22.2)	4 (23.5)	0.943
Overall revisions, *n* (%)	19 (42.2)	2 (22.2)	5 (29.4)	0.408
Effective EP	6 (13.3)	7 (77.8)	13 (76.5) 11 (64.7) for improved dumping 2 (11.8) for improved metabolic and dumping	** < 0.001**

An effective EP is indicated by the number of subjects who did not gain weight (< 10 kg) or with resolution of dumping symptoms over the total of subjects who completed follow-up

Values are presented as median with interquartile range (IQR) or absolute number with percentage in parentheses

Regarding postoperative outcomes, 63 (88.7%) patients could be discharged at the same day of the intervention; among the other 8, 6 stayed overnight and one patient was hospitalized 4 days after endoscopic clipping of an iatrogenic perforation. Another one with dysphagia had a hospital stay of 5 days because of a swollen anastomosis. (Table 3).

**Table 3:** Operative and postoperative results of endoscopic plication

Indication to EP Characteristics	Metabolic *n* = 45	Dumping *n* = 9	Metabolic and dumping *n* = 17
Size of the gastrojejunal anastomosis before EP, mm (IQR)	30 (25–30)	30 (25–30)	30 (30–30)
Size of the gastrojejunal anastomosis after EP, mm (IQR)	9.5 (8.5–10)	9.5 (9.5–10)	9.5 (8.9–9.5)
Size of the gastric pouch before EP, mm (IQR)	30 (30–50)	30 (20–30)	30 (30–40)
Number of sutures over the gastrojejunal anastomosis, *n* (IQR)	3 (2–3)	3 (2–3)	3 (3–4)
Operative time, min (IQR)	35 (32–50)	30 (25–43)	40 (36–48)
Perioperative complications			
Pain, *n* (%)	1 (2.2)	1 (11.1)	2 (11.8)
Stenosis, *n* (%)	1 (2.2)	1 (11.1)	2 (11.8)
Hematemesis, *n* (%)	1 (2.2)	0	1 (5.9)
Perforation treated endoscopically, *n* (%)	2 (4.4)	0	0
Perforation treated laparoscopically, *n* (%)	1 (2.2)	0	0
Size of the gastrojejunal anastomosis within 12 months, mm (IQR)	18 (15–20)	19 (16–20)	22 (15–29)

Values are presented as mean with standard deviation (SD). An effective EP is indicated by the number of subjects who did not gain weight (< 10 kg) or with resolution of dumping symptoms over the total of subjects who completed follow-up

### Weight loss outcomes

**Fig. 1:**
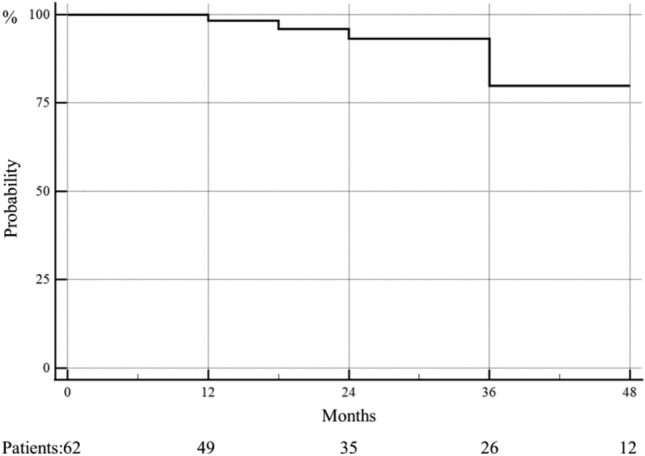
Patients with weight loss or weight maintained during follow-up

Weight loss assessment was carried out on 62 patients. Overall mean follow-up was 27.0 ± 19.7 months. Weight loss during follow-up (expressed in kg) is reported in detail in Table 4, and the analysis of patients who achieved weight loss or at least maintained weight over time is depicted in Fig. 1.

**Table 4:** Total weight loss (kg) during follow-up

Indication to EP Follow-up	Weight regain *n* = 45	Dumping *n* = 9	Weight regain and dumping *n* = 17
3 Months postop, kg (min–max)	− 4.8 (3.3) Effective 45/45	− 1.1 (2.1) Effective 9/9	− 4.1 (3.4) Effective 17/17
6 Months postop, kg (min–max)	− 4.9 (4.8) Effective 42/42	− 0.4 (1.0) Effective 9/9	− 3.5 (5.9) Effective 14/14
12 Months postop, kg (min–max)	− 3.3 (6.8) Effective 38/38	− 0.4 (4.0) Effective 8/8	− 2.6 (7.7) Effective 10/11
18 Months postop, kg (min–max)	− 1.9 (8.2) Effective 34/35	–	− 5.3 (4.6) Effective 7/8
24 Months postop, kg (min–max)	− 3.4 (9.5) Effective 27/29	–	− 5.7 (8.0) Effective 5/6
36 Months postop, kg (min–max)	− 0.4 (9.8) Effective 19/22	–	2.3 (11.9) Effective 2/4
48 Months postop, kg (min–max)	5.4 (11.2) Effective 8/10	–	9.7 (12.4) Effective 1/2

An effective EP is indicated by the number of subjects who did not gain weight (< 10 kg) or with resolution of dumping symptoms over the total of subjects who completed follow-up

Values are presented as mean with standard deviation (SD)

**Fig. 2:**
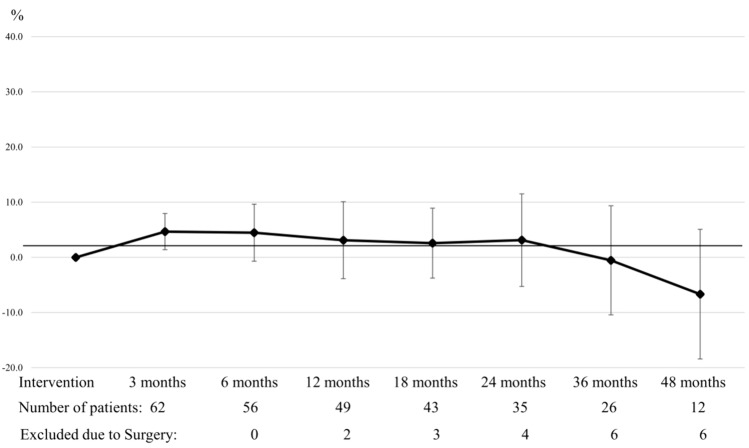
% Total Weight Loss during follow-up

The %TWL was 4.3% ± 3.4 within the first 3 months, 4.0% ± 5.0 at 6 months, 2.7% ± 6.8 at 12 months, 2.7% ± 6.2 at 18 months, 3.1% ± 8.2 at 24 months, − 0.6% ± 9.7 at 36 months and − 6.0% ± 11.5 at 48 months. (Table 5 and Fig. 2).

**Table 5:** Percentage of total weight loss (%TWL) during follow-up

Indication to EP follow-up	Weight regain *n* = 45	Dumping *n* = 9	Weight regain and dumping *n* = 17
3 Months postop, % (min–max)	4.8 (3.3) Effective 45/45	1.7 (3.6) Effective 9/9	4.4 (3.4) Effective 17/17
6 Months postop, % (min–max)	4.7 (4.6) Effective 42/42	0.7 (1.7) Effective 9/9	3.8 (6.6) Effective 14/14
12 Months postop, % (min–max)	3.2 (6.7) Effective 38/38	0.7 (6.2) Effective 8/8	2.7 (8.6) Effective 10/11
18 Months postop, % (min–max)	1.7 (6.6) Effective 34/35	–	5.7 (4.8) Effective 7/8
24 Months postop, % (min–max)	2.4 (8.3) Effective 27/29	–	6.2 (8.9) Effective 5/6
36 Months postop, % (min–max)	− 0.2 (9.4) Effective 19/22	–	− 2.5 (14.0) Effective 2/4
48 Months postop, % (min–max)	5.8 (11.9) Effective 8/10	–	− 11.2 (14.2) Effective 1/2

An effective EP is indicated by the number of subjects who did not gain weight (< 10 kg) or with resolution of dumping symptoms over the total of subjects who completed follow-up

Values are presented as mean with standard deviation (SD)

**Fig. 3:**
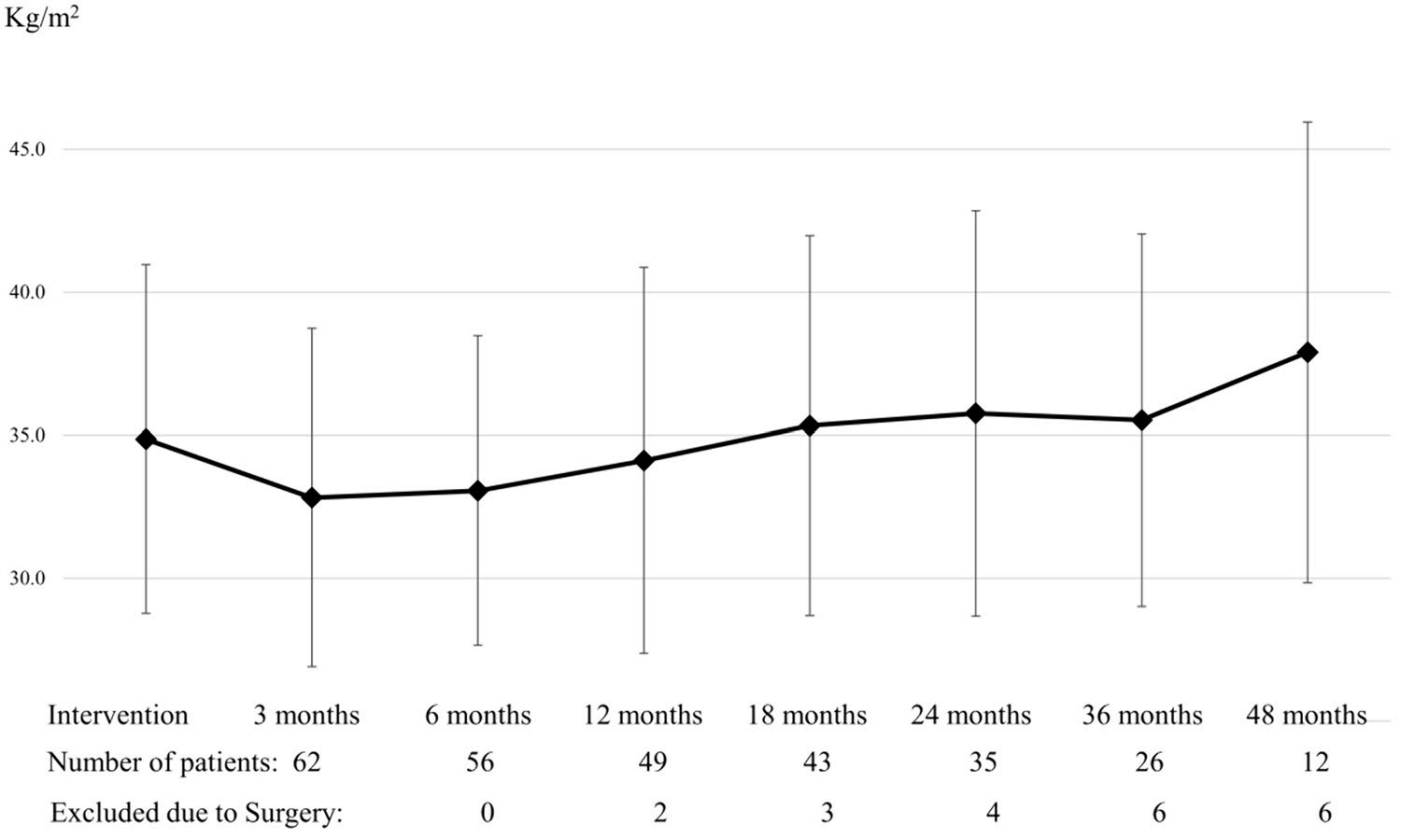
BMI during follow-up

The BMI change during follow-up was − 1.5 ± 1.2 kg/m^2^ within the first 3 months, − 1.4 ± 1.7 kg/m^2^ at 6 months, − 0.9 ± 2.3 kg/m^2^ at 12 months, − 0.9 ± 2.6 kg/m^2^ at 18 months, − 1.3 ± 3.2 kg/m^2^ at 24 months, 0 ± 3.4 kg/m^2^ at 36 months and 2.1 ± 3.8 kg/m^2^ at 48 months (Fig. 3).

### Remission of dumping syndrome

In 22 (84.6%) subjects out of 26 a persisting improvement of dumping syndrome was achieved. Three out of four subjects (75.0%) with insufficient improvement in symptoms were scheduled for surgery (gastric pouch resizing with minimizer ring) after a mean of 10.7 ± 3.6 months. During follow-up, 4 (18.2%) out of 22 subjects in whom EP was initially effective suffered a relapse. 3 (75.0%) of them underwent surgery (gastric pouch resizing with minimizer ring) after a median of 31.5 ± 26 months, the other one was planned for a revisional endoscopic procedure that was not successfully completed because of anesthesia complications.

## Discussion

Our study showed that TORe was effective in patients with dumping syndrome after RYGB. Perioperative complication rate was low and a significant weight loss in some patients and dumping resolution could be achieved during follow-up. Even as revisional intervention after failed surgical procedure for secondary weight regain TORe can be effective.

The present analysis represents data from one of the largest obesity and bariatric surgery centers in Switzerland with 300 procedures performed at three different sites per year. All patients undergoing an endoscopic procedure for secondary weight regain or unresolvable dumping syndrome or both between January 2015 and December 2020 were included. Although some studies have evaluated the effects of endoscopic revisions with different endoscopic suture systems and different techniques, long-term follow-up data is still scarce. Callahan has conducted a retrospective analysis on 70 subjects with weight regain after bariatric surgery [20]. The same endoscopic suturing system and equipment as in our study was used, with the difference that both interrupted, and purse-string sutures were applied. The mucosa was circumferentially ablated by argon plasma coagulation. An anastomotic diameter of 5–9 mm was achieved. These technical details are in accordance with our study, except for the suturing technique. In contrast to the Callahan cohort, in our subjects exclusively interrupted sutures were used. In the aforementioned study a % EWL of 14.9% after 1 year, 12.2% after 2 years 8.7% after 3 years, 3.2% after 4 years and 7% after 5 years was described. These results were slightly higher, at least in the early post-interventional course, compared to our cohort. Our subjects had an EWL of 11.4% at 12 months and at 2 years the EWL data did not differ so much: 8.2% in our cohort and 12.2% in the Callahan study. Long-term data still shows a positive effect after 5 years, but in our study the nadir weight loss was achieved 3 and 6 months after the intervention. Nevertheless, further weight regain can be avoided up to 3 years post-intervention and the Kaplan–Meier curve showed no weight regain after endoscopic intervention in more than 75% of our subjects, even after 48 months follow-up, which is comparable to the Callahan study.

Concerning post-interventional complications, a total of four complications were mentioned: two subjects with gastrointestinal bleeding (one occurring intraoperatively, and one requiring readmission due to melena). One subject was readmitted with obstruction, which resolved after nasogastric tube decompression. Another one was readmitted with perforation 1 day after the intervention and the lesion was sutured laparoscopically. The complication rate seemed to be comparable to our study, with a total of three perforations in 71 patients. Concerning the severity of complications in both the Callahan and our study, there was only one reported Clavien-Dindo IIIb, as two of the perforations in our cohort were recognized during the initial intervention and could be closed endoscopically with clips. Nevertheless, it is striking, that in our cohort the occurrence of perforations seemed to be higher while in the compared study the rate of bleeding was more frequent. Most subjects in the study mentioned above were discharged at the day of revision (85%, 57/67), which is comparable to our results (88.7%, 63/71). Nowadays almost all patients are discharged at the day of the intervention.

In another study, Tsai et al. analyzed 107 subjects with a mean follow-up time of 9.2 months. Most subjects suffered from secondary weight regain (*n* = 81), whereas 13 of them presented with dumping syndrome and further 13 with both dumping syndrome and weight regain [21]. The mean BMI before the intervention was 32.9 kg/m^2^ which is comparable to our cohort (mean BMI 34.5 kg/m^2^). They could demonstrate a mean weight loss of 8 kg after 12 months compared to a mean weight loss of 3.3 kg after the same time period in our cohort. The complete absence of complications is remarkable. With a mean follow-up of 9 months, the follow-up period was much shorter compared to our study. The authors reported that in 26 subjects a second TORe procedure was necessary and in 13 others a laparoscopic pouch revision was required. Technical details seemed to be analogous to our interventions. Tsai et al. routinely used 1 or 2 sutures with 4 to 6 stitches and primed the anastomosis with argon plasma coagulation. Two sutures resulted in better weight loss. However, long-term data is missing.

The treatment strategy was identical to the algorithm at our center. After 1 or 2 endoscopic procedures a surgical revision is recommended. Depending on the preoperative examinations and the intraoperative findings, a pouch resizing, a revision of the anastomosis or an implantation of a silastic ring is performed.

Dhindsa analyzed 850 subjects from 13 independent cohorts in a meta-analysis [22]. He reported a short-term weight loss of 6.14 kg, 10.15 kg, and 7.14 kg at 3, 6, and 12 months respectively compared to 4.8 kg, 4.9 kg, 3.3 kg at the same time points in our subjects. Results seemed to be slightly better than in our study group. The TWL (%) was more pronounced in the present meta-analysis compared to our study where a TWL of 6.69% vs.4.8% at 3 months, 11.34% vs. 4.7% at 6 months and 8.55% vs. 30.2% at 12 months was seen. The overall frequency of severe adverse events in TORe was rather low with a reported rate of 0.57% ± 1.35%. This rate was clearly lower than in our cohort (hematemesis *n* = 12.2%, and perforation treated endoscopically *n* = 36.7%).

### Dumping syndrome

In the present analysis a TORe has been performed in 26 subjects to treat dumping syndrome. Twenty-two subjects (84.6%) showed an improvement of symptoms. Four subjects showed recurrent symptoms and in three of them a revisional surgery was performed (pouch revision and implantation of a silastic ring). In accordance to our results, Tsai reported an initial success rate of 77.5% in 40 subjects whereas nine suffered from recurrence of dumping syndrome and needed repeated TORe (*n* = 7) and laparoscopic revision (*n* = 2) [23].

In a multicenter study, Vargas et al. reported 115 patients with dumping syndrome non-responsive to medical therapy. After endoscopic narrowing of the gastrojejunal anastomosis a significant reduction of Sigstad`s score and a very low failure rate in nine subjects (3%) was noticed. Subjects with recurrent symptoms underwent either another TORe for gastrojejunal anastomosis dilation (*n* = 3 out of 9) or an insertion of a feeding tube (*n* = 3 out of 9) [24].

Petchers et al. analyzed 98 patients which were treated with a TORe procedure using OverStitch™ after gastric bypass surgery for obesity and dumping syndrome. They reported a resolution rate of 88% and positive results of 84% remained even after a long-term follow-up of 3, 5 years on average. The effect of the TORe procedure remained in almost all subjects even years after the initial procedures. Only 7% of all cases needed a second procedure for recurrence of symptoms and endoscopic evidence of recurrent anastomotic dilation 2–3 years after the initial intervention [25].

### Limitations

This study has some limitations. First, follow-up intervals are inconsistent. Although we established a protocol for medical appointments, some subjects could not entirely respect it. The study design is retrospective; data quality depends on correct documentation and completeness of intervention variables, documentation of weight trajectories, and relief of dumping symptoms. Furthermore, the endoscopic procedure is not compared to a control group (e.g. conservative intervention through medical nutrition therapy, health coaching, physical activity, or a surgical reduction of the gastrojejunal anastomosis). Unfortunately, Sigstad`s dumping score or Arts` questionnaire were not assessed systematically to distinguish between early and late dumping syndrome. Finally, the size of the gastric pouch after the procedure was not recorded in our patient collective.

## Conclusion

TORe using an endoscopic suturing system is a safe and effective procedure in the treatment of patients with dumping syndrome after laparoscopic RYGB. A reduction in diameter of the gastrojejunal anastomosis is supposed to add restriction and thus to decrease the rate and velocity of food passage.

As we know from the era of gastric banding a pure addition of restriction is not successful in the long run. Further research should investigate whether TORe influences incretin levels in patients after secondary weight regain or dumping syndrome (e.g. MECCEO study currently conducted at our institution). Even years after an endoscopic procedure weight maintenance seems to be possible, although in some patients weight regain after 36 and 48 months occurs. From our data could be interpreted that TORe might be marginally effective on weight loss after 4-year follow-up. A prospectively randomized trial should be conducted to compare the effects of surgical methods like silastic banding of the gastrojejunal anastomosis or pouch resizing.
